# Innovations to Sustain Non-Communicable Disease Services in the Context of COVID-19: Report from Pakkred District, Nonthaburi Province, Thailand

**DOI:** 10.5334/gh.1003

**Published:** 2021-06-10

**Authors:** Soraphan Songsermpong, Sushera Bunluesin, Panisara Khomgongsuwan, Supattra Junthon, Danielle Cazabon, Andrew E. Moran, Renu Garg

**Affiliations:** 1 Pakkred Hospital, TH; 2 World Health Organization, TH; 3 Resolve to Save Lives, an Initiative of Vital Strategies, US; 4 Resolve to Save Lives, an Initiative of Vital Strategies, and Columbia University Irving Medical Center, US

**Keywords:** Non-communicable diseases, hypertension, diabetes, COVID-19, primary health care, Thailand

## Abstract

During the COVI9-19 pandemic, Pakkred hospital in Thailand implemented innovative practices to ensure the continuation of essential medical services for non-communicable disease patients. These practices included decentralized care, telemedicine, home blood pressure monitoring, community delivery of medicines, and facility infrastructure changes. Despite the decrease in hospital visits by hypertension patients during the pandemic, our results suggest that this package of interventions may have contributed to sustained hypertension and diabetes control rates in Pakkred district.

## Background

In early 2020, when COVID-19 was spreading globally, 59% of countries surveyed by a World Health Organization rapid assessment reported that access to outpatient non-communicable (NCD) services was restricted to a certain degree [1]. Public health systems faced the dual challenge of containing the outbreak while protecting health care workers (HCW) and maintaining routine services for hypertension, diabetes, and other chronic NCDs [1]. In Thailand, patients living with chronic diseases, especially elderly individuals and those with comorbidities, were unable or reluctant to visit health facilities for routine care. There were also challenges in maintaining NCD case management and screening programs for early detection of NCDs [2]. Outpatient visits decreased at a national level and in Bangkok [3]. This article aims to document innovative practices adopted by Pakkred Hospital of the Ministry of Public Health in Thailand to overcome challenges generated by COVID-19 and to assess whether they contributed to maintaining chronic care for patients with hypertension and diabetes.

## Health Care Context of Pakkred District, Thailand

Pakkred, a semi-urban district in the Nonthaburi Province of Thailand, had a population of 249,415 in 2019 [4]. The percentage of the population that is over 60 years old is 18.2%, and the majority have pre-existing chronic NCDs that require ongoing management. The numbers of patients on treatment for hypertension or diabetes in Pakkred Hospital are 5,881 and 3,015, respectively.

Since 2002, Thailand has provided universal health coverage through three public health insurance schemes. The health system in Pakkred consists of one public district hospital, two private hospitals, one university hospital, and eight health-promoting hospitals (HPHs). HPHs, each staffed by 3–5 nurses, are the first point of medical contact for the population and provide preventive and basic medical services, including hypertension and diabetes treatment. A doctor, nurse, pharmacist, and counselor from the district hospital visit weekly and oversee monthly NCD clinics at the HPHs. Pakkred is supported by approximately 240 community health volunteers (CHVs) who provide health promotion, disease prevention, and basic disease management services including chronic NCD care.

## Innovations to Deliver Chronic NCD Care During Covid-19

**Figure:**
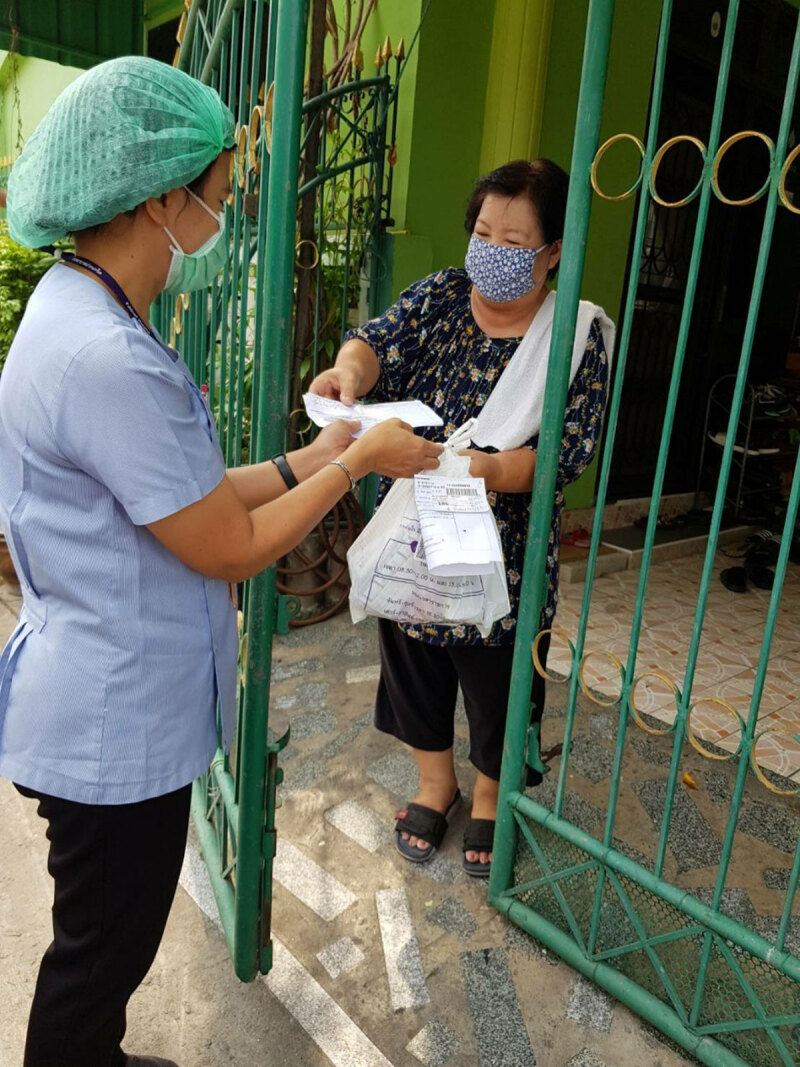
Home delivery of medication by hospital staff in Pakkred during the Covid-19 epidemic.

During Thailand’s COVID-19 pandemic, the health leadership of Pakkred Hospital prioritized maintaining essential services for patients with chronic diseases. As of March 16, 2020, several changes to primary health care were instituted in Pakkred District.

### Medication dispensing and delivery

After review of patient records by a nurse and doctor, patients with established diabetes or hypertension control were provided with repeat prescriptions and were given the option of having their medication delivered in the following ways:

- Pick-up of medications at district hospital and HPHs by healthier, younger relatives
- A drive-through facility at district hospital providing prescribed medications from a window
- Medication delivery through postal service by hospital staff
- Medication delivery by CHVs traveling via hospital van or motorcycle taxi

For patients with uncontrolled blood pressure (BP) or limited mobility, CHVs equipped with personal protective equipment measured BP at patients’ homes and transferred results to physicians, who adjusted prescriptions as needed.

### Facility infrastructure changes

Hospitals and HPHs implemented new measures to reduce COVID-19 exposure risk for patients with uncontrolled NCDs that still needed to visit facilities. Ventilation was improved, seating arrangements ensured physical distancing, additional outdoor patient waiting areas were implemented, number of patients indoors was restricted, and plastic screens were added as barriers at all points of contact with HCWs.

Additionally, a fast-track system was implemented for patients presenting to the hospital only for chronic NCD care. Patients received screening, measurement, consultation, and medications all in one place. For well-controlled patients, follow-up appointments were spaced 1–2 months longer during the peak COVID-19 period and staggered to reduce crowding at facilities.

### Technology for care and communication

To minimize unnecessary travel, LINE software text-messaging accounts were created for NCD clinics to enable online real-time or asynchronous consultations between hospital staff and patients, prescription refills, or medication pick-up or delivery. The LINE application also enabled 24/7 communication between doctors, nurses, CHVs, and home health care teams. A secure Facebook page was created for patient inquiries and to facilitate patient follow-up.

## Results

**Figure 1:**
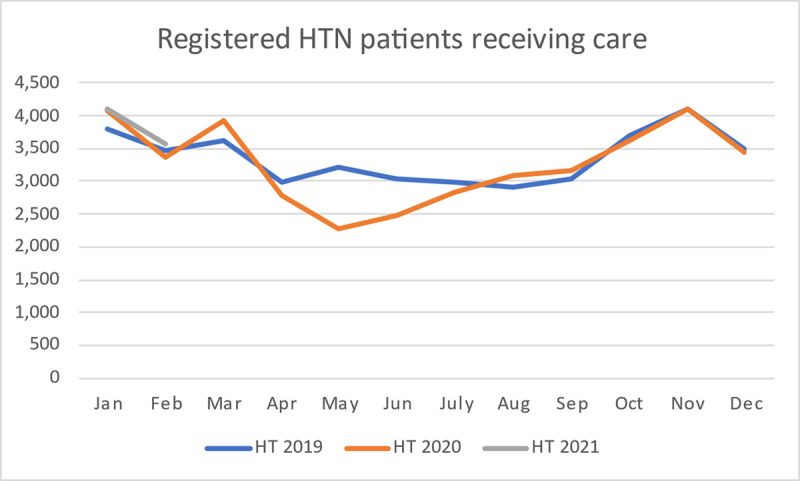
Registered hypertension patients on treatment and visiting OPD.

The number of patients with hypertension on treatment who visited outpatient departments (OPDs) at the hospital declined from March to May 2020, followed by a period of slow return to the hospital for care (Figure 1). By September 2020, the number of hypertension patients on treatment visiting hospital OPDs reached just over 3000, a number similar to one year prior, before the epidemic (September 2019). The hypertension control rate among all patients registered for treatment was maintained through the COVID-19 epidemic. Only 1% of patients could not be contacted for treatment or medicine refill. By September 2020, the hypertension control rate was 67% (3944/5,881), compared to 64% (3838/6055) in September 2019. The diabetes control rate was 24% (742/3079) in September 2019 and increased to 31% (941/3015) in September 2020.

Achievements during March–June 2020:

- Despite COVID-19 and the decrease in hospital visits by hypertension patients, a package of services including (i) adaptations to facility-based services, combined with (ii) outreach and community-based interventions may have contributed to sustained hypertension and diabetes control rates in Pakkred.
- Almost 70% of NCD patients had a relative collect medicine on their behalf; 25 cases of medicine pickup by relatives were by drive-through.
- 65 teleconsultations were performed via LINE.
- Medicine delivery by post was utilized 210 times.
- The fast-track system cut down NCD service time in HPHs from 2–3 hours to under one hour.
- No HCW and no NCD patient was diagnosed with COVID-19.
- Qualitative data reveals high overall patient satisfaction with the interventions.

## Conclusion

The response in Pakkred, Thailand, succeeded in delivering essential medical services to NCD patients during the Covid-19 epidemic. Several innovative practices initiated during the acute phase of the epidemic should continue in the long-term to improve patient-centered care for controlled NCD patients, including decentralized care, telemedicine, home BP monitoring, and community delivery of medicines. A pre-existing strong primary health care infrastructure, a network of CHVs, and a communications infrastructure provided the foundation for an innovative program to maintain essential NCD services.

## Data Accessibility Statement

Control rates are from the Health Data Center of the Ministry of Public Health, Thailand [5]. The outpatient department visit data are from the electronic health service record of Pakkred Hospital. This data is available upon reasonable request.
